# Application of bilingual simulated patients in the medical history collection for international medical students in China

**DOI:** 10.1186/s12909-023-04480-1

**Published:** 2023-07-22

**Authors:** Liping Zou, Juan Su, Jiao Li, Jing Wang, Jian Kang, Anning Yin, Haixia Ren, Xiaoda Jiang, Yijuan Ding, Ping An

**Affiliations:** 1 The Clinical Skill Center, Teaching Office of the First School of Clinical Medicine, Renmin Hospital of Wuhan University, 9 Zhangzhidong Road, Wuhan, 430060 Hubei Province China; 2 Department of Gastroenterology, Renmin Hospital of Wuhan University, 99 Zhangzhidong Road, Wuhan, 430060 Hubei Province China; 3 Key Laboratory of Hubei Province for Digestive System Disease, Renmin Hospital of Wuhan University, Wuhan, China; 4 Hubei Provincial Clinical Research Center for Digestive Disease Minimally Invasive Incision, Renmin Hospital of Wuhan University, Wuhan, China

**Keywords:** Simulate patient, Medical history collection, International medical student

## Abstract

**Background:**

In all international medical student (IMS) programs in China, language barriers between IMSs and Chinese patients greatly reduced the learning in clinical practice and brought great challenges to IMSs in their transition from preclinical to clinical practice. This study aimed to investigate the role of bilingual simulated patients (B-SPs) in IMSs learning of medical history collection in China.

**Methods:**

48 IMSs of grade 4 between October 2020 to Jan 2021 were enrolled in this study. During the training of medical history collection, students were randomly arranged into two groups trained with either B-SPs (B-SP group) or English-speaking SP (E-SP group). All SPs in Objective Structured Clinical Exam station (OSCE) were trained in the Affiliated Hospital of Wuhan University. Clinical skills in medical history collection were assessed by instructors during pre-clinical, post-clinical OSCE and clinical rotations.

**Results:**

The scores of IMSs in each group were analyzed in terms of medical history collection including the ability to effectively consult for information and key communication skills related to patient care. Our results indicated that IMS in B-SP group obtained similar scores in preclinical training for history collection (67.3 ± 8.46 vs 67.69 ± 8.86, *P* < 0.05) compared to E-SP group, while obtaining significantly higher score improvements between pre- and post-OSCE (17.22 (95% CI 12.74 to 21.70) vs 10.84 (95% CI 3.53 to 18.15), *P* = 0.0007).

**Conclusion:**

B-SPs are more conducive to doctor-patient communication and actually improve IMSs learning in medical history collection in China.

## Background

One of the consequences of globalization is the changing landscape of international higher education. The past two decades have seen a significant increase in the number of international students, that is, those who cross national borders in order to study. However, for these international those students always face a number of challenges including language and communication difficulties, cultural and educational barriers that affect their adjustment, socialization, and learning experience [1], psychological distress [2], or social isolation and immigration. Previous studies in the literature have identified foreign language proficiency and communication as predictors of students’ adaptation and well-being in different countries [2]. Recent reviews [3] [4] emphasized the language barrier and cross-cultural adaptation was one of the most commonly studied topics in international student studies with the rising number of international students in higher education worldwide.

Language barriers for international medical students (IMSs) may be more prominent because of the specialized and complex nature of medical vocabulary and higher requirements on the flexible use of language in doctor-patient communications. In the last two decades, China has experienced a dramatic rise in the enrolment of international students to its institutions of higher education, and clinical medicine is the most popular program for academic studies [5]. Most Chinese medical schools offer English-taught undergraduate program for IMSs in theoretical classes. However, during clinical rotations, language deficiency always becomes the main barriers for their whole health care delivery [5]. How to improve IMSs’ learning experiences for clinical practice arises more attention now. Bilingualism is a possible solution to the language barrier in IMS learning during clinical practice. However, researches on proper intervention of bilingualism in clinical practice for IMSs is still limited. Here, we focused on bilingual teaching in IMSs clinical practice and attempt to explore the effective ways of improving the clinical learning for IMSs.

Comprehensive and systematic medical history collection is one of the most important parts of clinical diagnosis, which is closely related to diagnostic strategy and provides unignorable safety information for clinical treatment. During the consultation, effective communication determines the harmonious start of patient-doctor interactions, which is related to patient satisfaction, compliance, and ultimately clinical outcomes [6–8]. Medical history collection training programs play an important role in medical curricula and are well recognized [9, 10].

Simulation patients (SPs) are a widely accepted tool for teaching communication skills with evidence of effective transfer into real life skills [11]. Through patient feedback on SPs, students’ communication skills, logical and systematic nature of inquiry and motivation are enhanced [12, 13]. In addition, peer-feedback and self-assessment contributed to a new understanding of future actions by students [14].

In recent years, there has been a dramatical increase in the number of IMSs receiving higher education, including clinical medicine, in China, especially from developing countries [10]. In their 6-year English-taught undergraduate program, clinical courses and related training were scheduled in year 4 and 5, while rotating clinical practice was in year 6 [5]. However, in all courses of the IMS program, language barriers between IMSs and Chinese patients greatly reduced their learning in clinical practice and was also a great challenge to their transition from preclinical to clinical. To date, few studies have provided practical solutions for preclinical and clinical training of IMSs. Considering the importance of language skills in clinical learning, we hypothesized that addressing language exchange and communication between IMSs and Chinese patients should be an effective intervention point to improve IMSs clinical learning outcomes, and incorporating bilingual applications in preclinical training of IMSs would provide a potentially practical solution for their clinical practice. In this study, we designed and evaluated the role of B-SPs in preclinical training and clinical practice for collecting medical histories. Before and after 3-month clinical rotation, their clinical skills in medical history collection were assessed by the scores in pre- and post-clinical OSCE and clinical rotations.

## Methods

### Participants

In October 2020, 48 fifth-year IMSs from 7 countries including Pakistan, Somalia, Kenya, Thailand, the United Kingdom, and South Korea from Wuhan University's six-year medical (English-taught) program were enrolled in this study. These IMSs attended a medical history collection lecture by the junior faculties. 3 B-SPs and 3 E-SPs without medical background attended a training seminar for SPs and the other 6 SPs attended the Objective Structured Clinical Examination Station (OSCE) and were qualified by the Wuhan University Affiliated Hospital. Two qualified OSCE examiners were also from Wuhan University. All study subjects consented and signed up to this study and all authors had access to the study data, reviewed and approved the final manuscript. This study protocol was approved by the Ethics Committee of the Wuhan University People's Hospital.

### Project description

#### Preclinical training and 1^st^ OSCE.

After lectures on medical history collection, all IMSs were randomized into two groups (B-SP group or E-SP group) in training workshops. The training sessions were conducted by a faculty member and assisted by 6 previously trained senior and chief residents. In E-SP group, English was used in the training. In B-SP group, SPs communicated with the IMSs in Chinese, and if IMSs found it difficult or unclear to understand, the Chinese was translated into English. All workshops were scheduled once a week and four simulation scenarios were applied. Each SP was given the same scenario description and learning objectives to interact in both groups.

After 1-month SP training, IMSs were randomly assigned to the OSCE (preclinical exercises), which were conducted by two qualified examiners from Wuhan University.

#### Clinical training

All IMSs then underwent a 3-month clinical rotation in the inpatient units (including gastroenterology, cardiology, and gastroenterology). The clinical faculties assessed the IMSs' performance in patient history collection and their ability to communicate verbally and nonverbally in clinical practice. Scores represent the average assessment of the three rotations.

#### 2^nd^ OSCE.

**Fig. 1:**
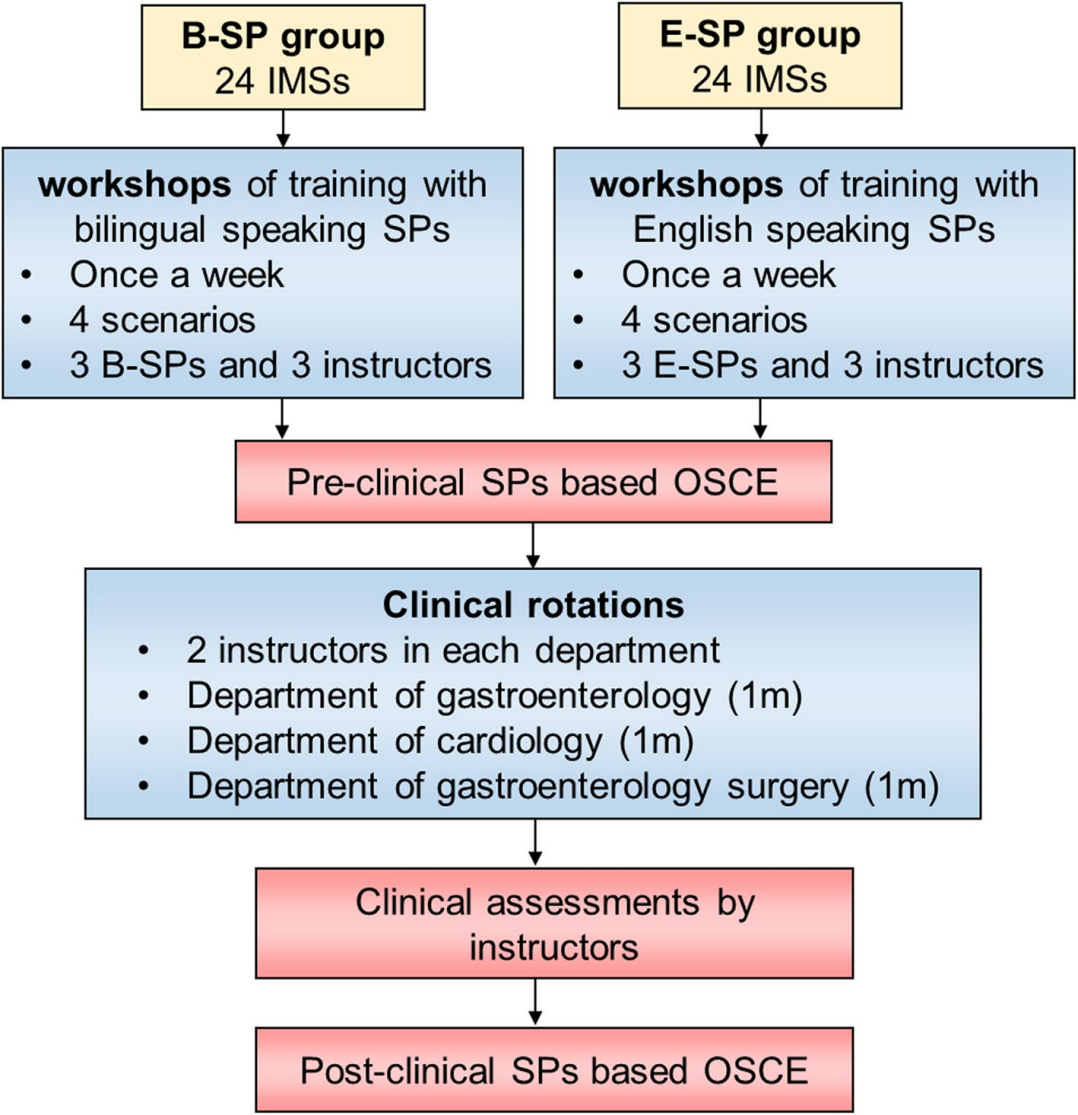
Research participants’ assignment flow diagram.

After clinical rotations, IMSs attended a second clinical skills OSCE administered by the same examiners as in the first OSCE assessment. English was used in both OSCE assessments. A flowchart of the researcher's tasks is shown in Fig. 1.

#### Simulation scenarios

Simulation scenarios were designed by senior physicians with extensive clinical and teaching experience in Renmin Hospital of Wuhan University. The scenarios were set up to take into account the culture differences between east and west and the actual situation of Chinese patients, simulating outpatient and ward situations as much as possible. The training programs focused on IMSs’ clinical analysis skills, communication skills and verbal and non-verbal humanistic care during the medical history-taking consultation process. All cases were discussed by experts and then officially allowed to be used in the training.

#### SP training

SPs should be “standardized” in their performances so students could get the same testing experience [15]. SPs were trained in history taking one week prior to IMSs workshop and OSCE. The training focused on basic medical knowledge, general examination in clinical skills, counseling, doctor-patient communication skills, introduction to OSCE, and the use of SPs in OSCE. Any medical information was provided by the SP only when asked by the IMSs and SPs responded without unnecessary explanations. Role-play exercises were conducted in hospital scenarios. B-SPs and E-SPs were trained in four medical history collection scenarios during a 2-day workshop with three experts in clinical skills and communication. Training was also conducted for all SPs for OSCE, uniform scoring criteria, and medical records. Only qualified SPs from Wuhan University participated in teaching and assessment tasks.

#### Evaluation of the IMS capabilities of medical history collection

The evaluation criteria for medical history collection included pre- and post-clinical OSCE evaluations. Ten aspects include self-introduction, appearance and etiquette, organizational arrangements, consultation content, information collection, respect for understanding, communication skills, citation verification, summary and end of consultation. The full score was 100 points. Each examiner used the same scoring criteria to assess IMSs performance.

For evaluation of the 3-month clinical rotation, clinicians from each of the 3 departments evaluated IMSs skills of medical history collection with Chinese patients at the end of rotations.

### Statistical processing

SPSS19.0 statistical software was used for data analysis. The data were expressed as mean ± standard deviation. After testing for normal distribution and homogeneity of variance, comparison between groups were analyzed by paired t-test. As for the differences of score changes between pre- and post-clinical OSCE, independent t-test was used. *p* < 0.05 indicates that the difference is statistically significant.

## Results

### IMSs characteristics at baseline

In this study, 48 IMSs were trained in B-SP- or E-SP-based training of medical history collection through evaluation by pre- and post-clinical OSCE and clinical instructors in clinical rotations. The undergraduate medical students were statistically balanced in terms of mean age, gender, and HSK level in Chinese (Table 1).

**Table 1:** Baseline IMSs characteristics

	**B-SP** **(*n* = 24)**	**E-SP** **(*n* = 24)**	***p* value**
Age (years, IQR)	22.4 (21–23)	22.6 (21–23)	0.5161
Age (years, IQR)	8/16	7/17	0.8271
HSK* levels of Chinese	4.21 ± 0.92	4.08 ± 0.99	0.9321

^*^HSK Hanyu Kouyu Kaoshi (Chinese level exam), *IMS* International medical student

### Assessment in preclinical OSCE (first OSCE) indicated that after preclinical training by B-SPs or E-SPs, IMSs had similar capability in medical history collection

IMSs medical history collection skills were assessed based on the ability to effectively consult for information and competency in key communication skills related to patient care: self-introduction, appearance and etiquette, organization, obtaining information with effective questioning skills, consultation content, behaviors that demonstrate caring and humility, communication skills, providing evidence through effective skills, summarization, and consultation closure.

We first analyzed the effect of preclinical training by B-SPs or E-SPs on IMS learning for medical history collection (preclinical OSCE). In this preclinical OSCE, no significant differences between two groups were found regarding to scores of skills in gathering information from patients, communication skills, organization, and summarization after the preclinical session trained with B-SPs and E-SPs (Table 2). Similarly, scores for self-introduction, closing consultation, and effective questioning skills did not differ between two groups. IMSs in both groups performed similarly in patient care. Compared to E-SP group, IMSs in B-SP group had the same clinical skills and achieved similar total scores for both verbal and nonverbal skills [67.3 (95% CI 63.9 to 70.7) vs 67.69 (95% CI 64.1 to 71.2), *P* = 0.8767].

**Table 2:**
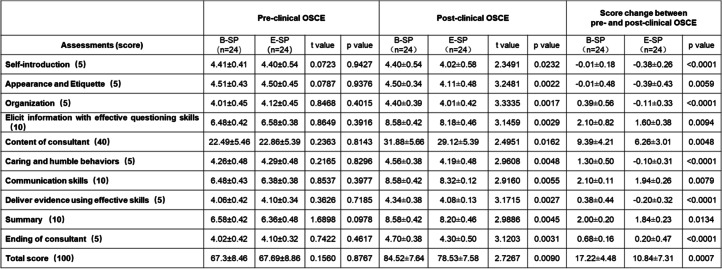
Pre- and post-clinical scores of medical history collection in OSCE

### Assessment in clinical rotations revealed that IMSs in B-SP group achieved more improvement in medical history taking

Interestingly, during the clinical rotations, IMSs in B-SP group improved their ability to communicate with Chinese patients and their skills in collecting medical histories were significantly enhanced. IMSs trained by B-SPs had a more appropriate approach when communicating with Chinese patients. Clinical instructors also rated significantly higher for IMSs in B-SP group than that in E-SP group in history collection [77.83 (95% CI 74.3 to 81.3) vs 70.97 (95% CI 67.4 to 74.5), *p* < 0.01] (Table 3). IMSs in B-SP group appeared to be more comfortable and interested in communicating with Chinese patients while IMS in E-SP group had more challenges in patient communication. These results indicated that bilingual SPs-based training helped IMSs to overcome language barriers and facilitated their clinical studies.

**Table 3:** Clinical scores of medical history collection during clinical rotations

Assessments (score)	B-SP (*n* = 24)	E-SP (*n* = 24)	*t* value	*p* value
Self-introduction(5)	4.44 ± 0.39	4.01 ± 0.58	2.7589	0.0083
Appearance and Etiquette(5)	4.51 ± 0.43	4.10 ± 0.58	2.7819	0.0078
Organization(5)	4.40 ± 0.41	4.01 ± 0.45	3.1385	0.0030
Elicit information with effective questioning skills(10)	7.48 ± 0.42	7.20 ± 0.28	2.7175	0.0092
Content of consultant(40)	28.49 ± 5.46	24.86 ± 5.59	2.5266	0.0150
Caring and humble behaviors(5)	4.46 ± 0.48	4.11 ± 0.41	2.7162	0.0093
Communication skills(10)	7.48 ± 0.43	7.18 ± 0.28	2.8642	0.0063
Deliver evidence using effective skills(5)	4.46 ± 0.42	4.12 ± 0.43	2.7711	0.0080
Summary (10)	7.58 ± 0.42	7.20 ± 0.44	3.0605	0.0037
Ending of consultant(5)	4.56 ± 0.42	4.18 ± 0.46	2.9886	0.0045
Total score(100)	77.83 ± 8.80	70.97 ± 8.86	2.6912	0.0099

### Assessment in post-clinical OSCE (second OSCE) showed that after the clinical rotations, IMSs in B-SP group had a greater improvement in their ability to collect medical history

Medical history collection is one of the most important parts of clinical skills and requires students to obtain important history data through effective communication with patients in a limited time. Students should train to establish a good doctor-patient relationship and to fully reflect humanistic care in their communication. After clinical rotations, IMSs in both B-SP and E-SP groups improved their ability to collect medical history [84.52 (95% CI 81.5 to 87.6) vs 67.3 (95% CI 63.9 to 70.7)] in the B-SP group; 78.53 (95% CI 75.5 to 81.6) vs 67.69 (95% CI 64.1 to 71.2) in the E-SP group, when compared with preclinical OSCE, respectively] (Table 2). However, the score changes of IMSs in B-SP group were much higher than E-SP group [17.22 (95% CI 12.74 to 21.70) vs 10.84 (95% CI 3.53 to 18.15), *P* = 0.0007). These results clearly indicated that bilingual history collection training is a practical way to address the learning problems of IMSs in clinical practice and thus improve their clinical skills competency.

## Discussion

The majority of IMSs in clinical medicine at Chinese universities come from developing countries and their English proficiency is comparable to that of students from countries where English is their native language. During their 6 years of medical studies, all courses were organized in English. Although these IMSs learned Chinese prior to clinical practice and lived in China for at least 3 years, they still had difficulties in some of their interactions with Chinese people, especially during their medical studies in China. In a real clinical history collection practice, IMSs need to communicate directly with Chinese patients and conversing with fluent Chinese becomes critical. Otherwise, IMSs will lack in-depth experience in clinical practice. Here, we aim to reveal whether bilingual SPs in preclinical training provide a practical way to improve the clinical skills of IMS in history collection at Chinese universities.

In China, medical courses taught in English were arranged for IMSs, and training in clinical skills with English-speaking SPs was also required. The training with SPs provided adequate and necessary information for IMSs. During the simulated communication exercises, IMSs were adequately prepared for further clinical practice. Our study indicated that preclinical training with the application of B-SPs did not show a more significant improvement in IMS history taking skills during the first OSCE (preclinical) evaluation. There was no significant difference between E-SP and B-SP groups in the skills of collecting patient information in terms of counseling, doctor-patient communication skills, organization and summarization. These findings suggested that preclinical use of B-SPs had not a prominent impacts for IMS in medical history collection before their clinical rotations. Its role in clinical rotations and clinical practice required further exploration.

For Chinese patients, they do not use English. This language barrier clearly limits the learning of IMSs when it comes to actual communication with our clinical patients. Even though IMSs have lived in China for 3–4 years and have studied Chinese for a long time, they still find it difficult to communicate with Chinese patients. The main reasons for this include: (1) IMS were not familiar with Chinese patients' expression habits and logical ways of thinking; and (2) IMS lacked Chinese communication training when collecting medical histories. In our study, IMSs trained with B-SPs rather than E-SPs during clinical rotations were better suited to communicate with Chinese patients. IMSs in B-SP group had significantly fewer difficulties due to language barriers than E-SP group. Clinical instructors also rated history collection significantly higher for IMSs in B-SP group than that in E-SP group. IMSs in B-SP group appeared to be more interested in communicating with Chinese patients. In contrast, for IMSs in E-SP group, they were usually unable to communicate effectively with Chinese patients. Clinical instructors, IMSs, and Chinese patients often had to communicate repeatedly, and the language barrier brought more difficulties for IMSs. The training of bilingual SPs helped IMSs overcome the language barrier to benefit their clinical studies.

The higher levels of medical history taking skills of IMSs in B-SP group were further confirmed by the second OSCE (post-clinical). Although IMSs in both B-SP and E-SP groups improved their ability to collect medical history compared with their scores in the first OSCE, respectively, the score changes between two OSCEs for IMSs in B-SP group were much higher than that in E-SP group. These results further verified that B-SPs-based preclinical training was a feasible solution to the learning problems of IMSs in clinical practice, thus facilitating the improvement of their clinical skills.

Our results and conclusions have important implications for the clinical teaching for international medical students in other countries. The following aspects should be noted in clinical bilingual teaching for international medical students. Firstly, B-SPs who are native English speakers should have good communication skills in Chinese, especially in medical Chinese, and have more familiar knowledge of Chinese culture. Secondly, B-SPs and E-SPs should not have related medical background which will guarantee the real situation of patient scenarios. All qualified SPs should attend a training seminar for this SP training. Furthermore, B-SPs and E-SPs in preclinical training should not attend OSCEs. Finally, SPs for twice OSCEs should be the same batch which could ensure the consistency of same assessment criteria.

## Conclusion

In conclusion, the application of B-SPs has improved the medical history collection ability of IMSs. It not only alleviates the language barrier for medical students in learning and training, but also improves students' clinical practice and facilitates their clinical skills in history taking, thus improving the quality of teaching and providing a good method for bilingual courses in other medical majors. The training of B-SPs has high requirements for teachers and students, including good language skills, performance and imitation skills, short training basic medical knowledge afterwards. All these need to be further explored and improved in the future.

## Provenance and peer review

Not commissioned; externally peer-reviewed.

## Data Availability

The data underlying this article will be shared on reasonable request to the corresponding author.

## Abbreviations

B-SPs: Bilingual simulated patients
IMSs: International medical students
E-SPs: English-speaking simulated patients
OSCE: Objective Structured Clinical Exam station
